# Ultra-Broadband Wearable Antenna with Thermal Sensitivity Based on Surface-Modified TiO_2_-PTFE-PDMS Nanocomposites

**DOI:** 10.3390/mi16060629

**Published:** 2025-05-27

**Authors:** Baoli Mi, Qingya Meng, Junping Duan, Bowen Su, Ma Jian, Yangyi Shi, Binzhen Zhang

**Affiliations:** 1 State Key Laboratory of Extreme Environment Optoelectronic Dynamic Measurement Technology and Instrument, North University of China, Taiyuan 030051, China; b200613@st.nuc.edu.cn (B.M.); duanjunping@nuc.edu.cn (J.D.); 13015475931@163.com (B.S.); m821207588@163.com (M.J.); 18382456984@163.com (Y.S.); 2 School of Instrument and Electronics, North University of China, Taiyuan 030051, China; 13315684479@163.com

**Keywords:** wearable, KH570, modification, ultra-wideband, SAR

## Abstract

In this study, a composite substrate with adjustable dielectric properties was prepared, and its promising application in wearable medical device antennas was demonstrated. 3-Methacryloxypropyltrimethoxysilane (KH570) was used to modify titanium dioxide (TiO_2_) nano-powder, and the modified powder was blended with a mixture of polydimethylsiloxane (PDMS) and polytetrafluoroethylene (PTFE) under the action of anhydrous ethanol. The resulting polymer material had the advantages of hydrophobicity, softness, low loss, and a high dielectric constant. Meanwhile, the effects of the KH570 mass fraction on the microstructure and dielectric properties of TiO_2_-PTFE-PDMS composites were investigated, and the results showed that when the mass fraction was 5%, the composites exhibited better dielectric properties in the range of 2–12 GHz. Finally, an ultra-wideband antenna with an operating frequency band in the range of 2.37–11.66 GHz was prepared based on this composite substrate. The antenna demonstrated significant potential for future applications in detecting environmental thermal changes due to its special temperature-sensitive linear frequency shift characteristics, and its effect on the human body under bending conditions was studied. In addition, specific absorption rate (SAR) measurements were performed to assess the effects of antenna radiation on the human body in practical applications.

## 1. Introduction

In response to the poor mobility and inefficiency of medical personnel in traditional patient care settings, the use of wearable medical devices has not only greatly improved hospital care services but also saved the time of medical personnel. Antennas play a crucial role in establishing wireless communication between wearable medical devices, which have the ability to monitor the wearer’s biosignals and communicate these data with the environment in order to provide continuous information about a person’s state of health [1,2]. Conventional antennas based on rigid substrates have limited application in wearable devices, since the basic daily activities of patients cause various physical deformations in these antennas [3,4]. In addition, wearable antennas serve as devices that radiate and receive electromagnetic waves for body-centric communication systems [5,6,7], and we must consider their safety risks to the human body in long-term electromagnetic radiation environments [8]. The above limitations pose significant challenges for the design of wearable antennas.

With the development of modern wireless communication technology, the widespread application of patient-vital-sign-monitoring systems in clinical medicine has led to increasing demand for wearable antennas, and achieving high-quality transmission of signals with minimal size while ensuring comfort has become a major challenge. It is well known that the dielectric properties of dielectric substrates have an important impact on antenna performance. A low loss tangent can reduce signal attenuation and distortion and improve the antenna’s transmission efficiency and quality [9,10], while a relatively high dielectric constant can reduce the antenna size to make the antenna structure more compact [11]. Recently, many new composite substrate materials have been developed by researchers for antenna design. For example, Vilesh et al. [12] used hot-pressed silicone rubber and low-loss BaBiLiTeO_6_ ceramics to fabricate a composite material, suitable for 5G communication systems, which can be used as a substrate for microstrip patch antennas in the high-frequency range. L. Catarinucci et al. [13] proposed using alumina oxide and barium titanate as ceramic fillers, and they synthesized ceramic-doped organosilicon structures by 3D printing molds, which offer the advantages of flexibility, conformability, and comfort. Elmobarak et al. [14] prepared flexible composite antennas with radiation efficiency above 70% by embedding highly conductive fabrics into thin composite laminates made of E-glass fiber mats and epoxy resins via an impregnation process. Compared to textiles for which the dielectric properties are not readily modifiable [15,16,17], polymer composites provide a wider range of optimization options, as the dielectric properties of the composites can be modified by filling them with functional fillers [18,19,20,21]. However, due to the difference in surface energy between nanofillers and the polymer matrix [22], direct physical blending is highly susceptible to the agglomeration phenomenon, which affects the dielectric properties of the composites [23]. Therefore, in this paper, surface modification of a doped TiO_2_ filler using a silane coupling agent (3-methacryloxypropyltrimethoxysilane, KH570) is applied to improve its dispersion state in a PDMS/PTFE blend and enhance the antenna’s substrate performance.

In this paper, a wearable ultra-wideband antenna is fabricated based on TiO_2_-PTFE-PDMS composites with an impedance bandwidth ranging from 2.37 GHz to 11.66 GHz under flat conditions and an SAR compliant with wearable standards. The dielectric properties of the dielectric substrate are enhanced through the chemical modification of TiO_2_ by KH570. At the end of this article, the details of the fabrication process for the antenna are given and the test results are presented.

## 2. Materials and Methods

### 2.1. Preparation of Substrate

#### 2.1.1. Modification of Nano-TiO_2_ Powder

**Figure 1:**
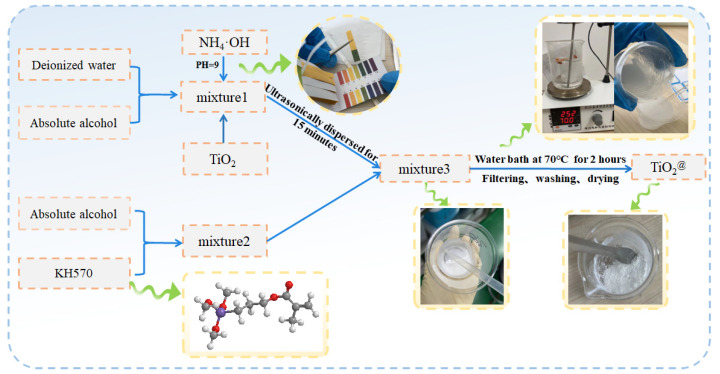
Flow chart of nano-TiO_2_ modification process.

A mixture of 20 mL of anhydrous ethanol and deionized water was prepared according to a ratio of 1:1 by volume, and the pH of the mixture was adjusted to 9 by adding ammonia water with a mass fraction of 25%. Then, we weighed 5 g of dry TiO_2_ powder, added it to a beaker containing the mixture, and then stirred it for 20 min in a constant-temperature water bath at 70 °C so that the TiO_2_ powder was uniformly dispersed in the mixture. Next, 10 mL ethanol solution containing 0.05 g KH570 (the weight ratio of KH570/TiO_2_ varied from 1% to 10%) was slowly added to the above mixture and reacted for 1.5 h under rapid stirring at a constant temperature in a water bath. Finally, the reaction mixture was filtered, washed with ethanol several times, filtered again, and dried for 24 h in a constant-temperature drying oven at 60 °C. Then, the modified TiO_2_ powder was obtained. The modification process is shown in Figure 1.

#### 2.1.2. Preparation of Composite Dielectric Substrate

**Figure 2:**
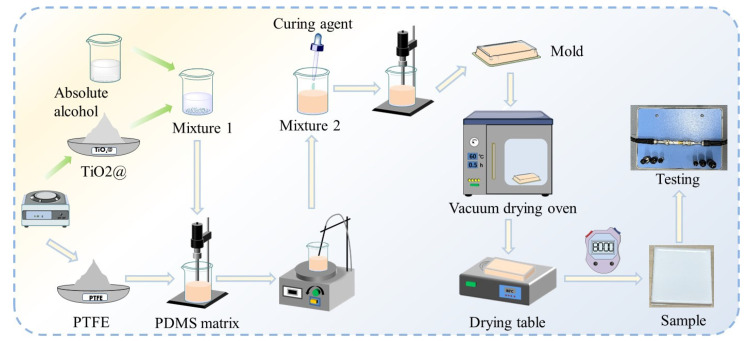
Flow chart of composite dielectric substrate preparation.

Due to the poor grinding of the modified titanium dioxide powder, the relatively large particles mixed into PDMS would cause a bottling phenomenon, which would adversely affect the performance of the composite substrate [24,25,26,27,28,29]. The solution blending method using anhydrous ethanol as the solvent can effectively solve this problem. The specific procedure is as follows: A quantity of 0.35 g of modified TiO_2_ is mixed with 2 mL of anhydrous ethanol in a beaker. The mixture is stirred thoroughly with a glass rod until no visible particles remain. Subsequently, 1.15 g of PTFE and 3.5 g of PDMS (PDMS/TiO_2_/PTFE = 70%:7%:23%) are added, followed by ultrasonication for 30 min to ensure uniform dispersion of the components. Then, the thermostatic magnetic stirrer is heated in a water bath at 70 °C for 4 h to ensure complete volatilization of the anhydrous ethanol. The curing agent is added (the mass ratio of PDMS to the curing agent is 10:1), and the mixture is stirred with a magnetic stirrer for 20 min (at a speed of 300 rpm); then, it is poured into a mold and dried in a vacuum drying oven for 30 min for defoaming. Finally, it is transferred to a baking table for curing (at 80 °C for 80 min). A flow chart of composite dielectric substrate preparation is shown in Figure 2.

### 2.2. Performance Testing

#### 2.2.1. Infrared Spectral Testing

**Figure 3:**
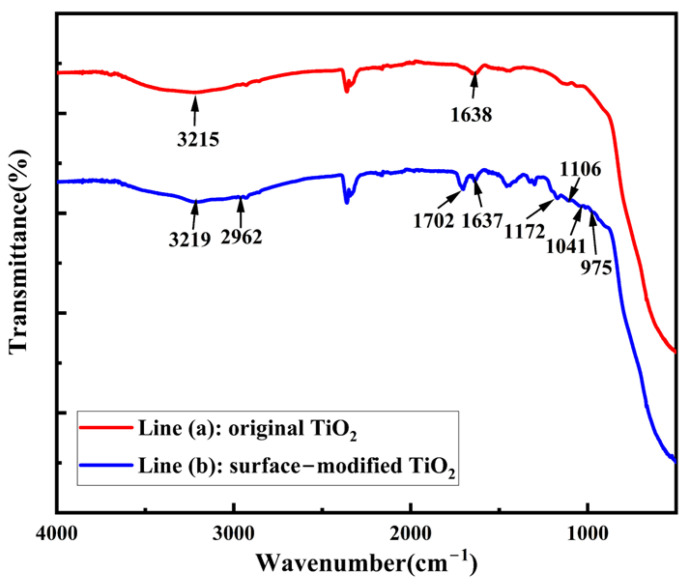
Infrared spectra of titanium dioxide before and after surface modification.

From the comparison of spectral lines (a) and (b) in Figure 3, it can be seen that the TiO_2_ nanoparticles before and after modification had characteristic spectral bands of hydrophilic -OH groups at 3215 cm^−1^ and 3219 cm^−1^, respectively. Due to the adsorption of a large amount of water on the surface of the TiO_2_, the peaks are broad.

Moreover, the spectral line (b) produced a red-shift phenomenon owing to the formation of intermolecular or intramolecular hydrogen bonds. The absorption peaks between 800 and 500 cm^−1^ in Figure 3 belong to the characteristic peaks of TiO_2_. As line (b) shows in Figure 3, the peaks at 2962 cm^−1^, 1702 cm^−1^, and 1637 cm^−1^ correspond to the C-H, C=O, and C=C stretching vibration peaks, respectively, and the C=O band is stronger than the C=C band. The Si-O-Si bond appears at 1172 cm^−1^ [30,31], representing the condensation reaction between silanols. The absorption peaks at 1106 cm^−1^ and 1041 cm^−1^ are caused by O-Si asymmetric flexible vibration. The peak at 975 cm^−1^ is characterized by the tensile vibration of Ti-O-Si, indicating that the Si-O-C on the surface of KH570 reacts with the hydroxyl groups on the surface of titanium dioxide. Therefore, the silane coupling agent was successfully grafted onto the surface of the titanium dioxide nanoparticles.

#### 2.2.2. Dielectric Property Testing

**Figure 4:**
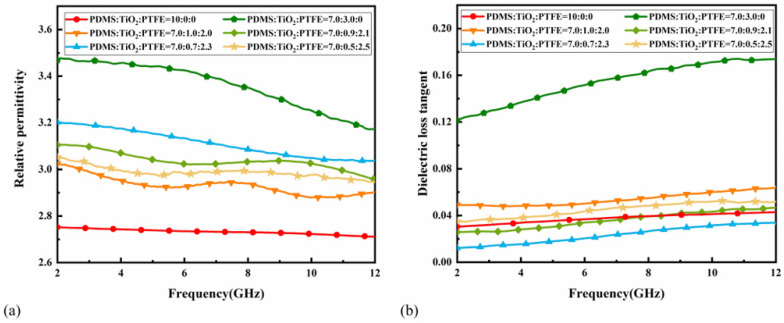
(**a**) Dielectric constant test results for TiO_2_-PTFE-PDMS composites with different doping ratios. (**b**) Loss angle tangent test results for TiO_2_-PTFE-PDMS composites with different doping ratios.

The dielectric properties of the composite dielectric substrate were tested using a vector network analyzer (Keysight 85054D, made by Keysight Technologies, Santa Rosa, CA, USA) with a coaxial dielectric probe kit and sequentially calibrated using open, short, and loaded calibrators to minimize the measurement errors. The final measured dielectric properties of the TiO_2_-PTFE-PDMS composite substrate with frequency for different doping ratios are shown in Figure 4a,b. The low dielectric constant and high loss of single PDMS are shown by red lines; these properties are not conducive to the miniaturization of antenna size [32]. The dielectric properties can be controlled by adjusting the proportion of doped materials.

**Figure 5:**
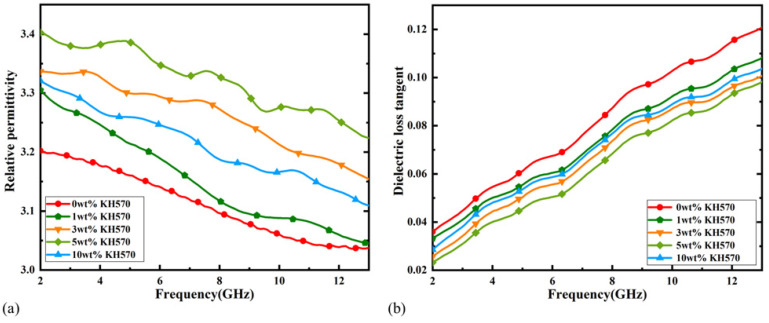
(**a**) Dielectric constant versus frequency for TiO_2_-PTFE-PDMS composites modified with different volume fractions of KH570. (**b**) Loss angle tangent versus frequency for TiO_2_-PTFE-PDMS composites modified with different volume fractions of KH570.

Figure 4a,b shows that when PDMS/PTFE/TiO_2_ = 7.0:0.7:2.3, the composites had better dielectric properties; hence, this ratio was used for further studies. In order to demonstrate the effect of KH570 on the composites at this ratio, TiO_2_-PTFE-PDMS composites with different volume fractions of KH570-modified TiO_2_ filler were prepared. From Figure 5a,b, it can be seen that the modified composite material had a higher dielectric constant, which increased with an increase in the content of coupling agent KH570. When the coupling agent content was increased to 10%, the dielectric constant of the composite material decreased, while the loss tangent showed the opposite trend. The final results show that the composites containing 5 wt% KH570 had better dielectric properties.

#### 2.2.3. SEM Characterization

**Figure 6:**
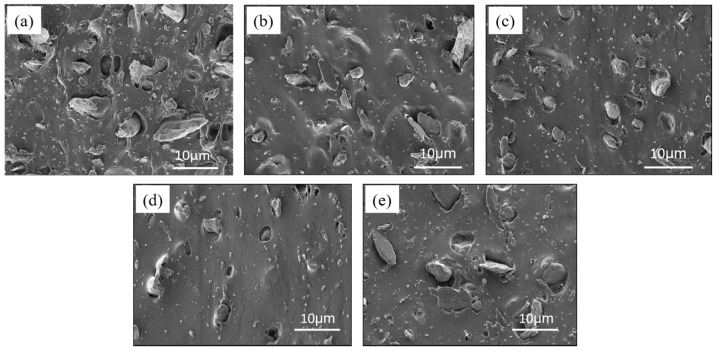
Cross-sectional SEM diagram of TiO_2_-PTFE-PDMS composites modified with KH570 coupling agent doped with different mass fractions: (**a**) 0 wt%; (**b**) 1 wt%; (**c**) 3 wt%; (**d**) 5 wt%; (**e**) 10 wt%.

A bulk composite sample was adhered to a conductive adhesive and sprayed with gold using a Quorum SC7620 sputter coater for 45 s, for which the gold spraying current was 10 mA. Then, the sample morphology was observed under the scanning electron microscope of a TESCAN MIRA LMS operating at an acceleration voltage of 3 kV. The final scanning electron microscope images are shown in Figure 6. By observing the cross-sectional SEM images of TiO_2_-PTFE-PDMS composites, it can be seen that without the coupling agent, the volume of filler particles inside the composite material was large, the internal pores were obvious, and the PDMS failed to wrap the filler well. As the coupling agent content increased, the filler size decreased, and it existed mainly in the form of small particles. In addition, the surface of the composites became flatter and smoother, with better overall dispersion. However, when the content of coupling agent was increased to 10%, there were more obvious large particles, probably because of the polymerization reaction involving the excess coupling agent. This caused the amount of coupling agent grafted on the surface of the titanium dioxide to decrease, so its compatibility with PDMS became worse.

### 2.3. Antenna Structure Design

The circular disk monopole antenna (CDM) is a commonly used UWB antenna with a simple structure, compact size, and excellent broadband performance. The broadband performance of this antenna is further improved by cutting rectangular and circular slots on the circular disk-shaped patch. High-frequency currents are distributed along the edge of the disk, generating electromagnetic radiation. This design reduces the radiation efficiency loss of the antenna in the high-frequency band and optimizes the performance throughout the entire frequency band. With the development of communication technology, the circular disk monopole antenna has evolved from the traditional circular shape into various shapes such as the umbrella shape [33] and Mickey Mouse [34]. Compared with that of the traditional circular disk monopole antenna, the effective length of the proposed guitar-shaped antenna’s radiator is increased by adding four circular arc curves at the edge of the radiation patch and cutting rectangular slots, without affecting the simplicity and compactness of the CDM structure. The disturbed surface current path length is increased. Bending is introduced at the corners, away from the feed line that minimizes the ground-plane radiation. With its compact size and excellent broadband performance, the guitar-shaped antenna is suitable for wireless communication devices that require miniaturization and high performance. It has broad application prospects in wireless local area networks (WLANs), wireless personal area networks (WPANs), the Scientific and Medical (ISM) band, and other frequency bands [35].

**Figure 7:**
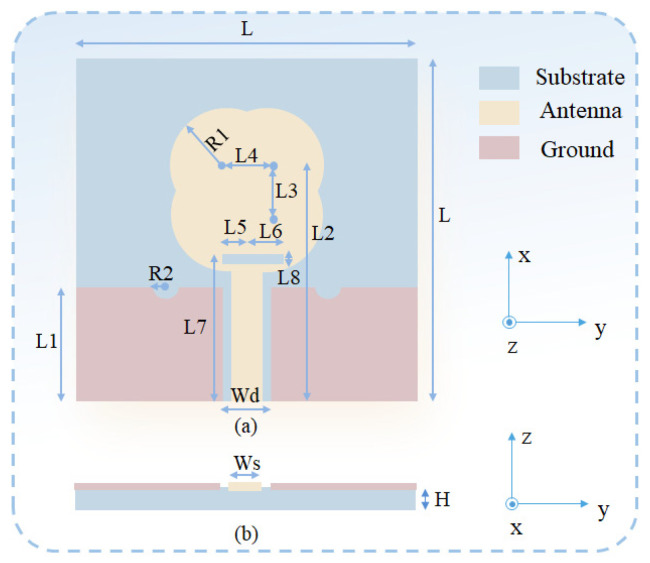
The antenna geometry: (**a**) front view; (**b**) side view.

The geometry of the ultra-wideband wearable antenna designed in this paper is shown in Figure 7, with a shape similar to that of a guitar and dimensions of L × L × H. The substrate is doped with composite material, which is highly flexible, and its dielectric properties are shown in Figure 4. The coplanar waveguide feed used in the antenna simplifies the fabrication process and reduces the fabrication cost at the same time. Most importantly, the coplanar waveguide feed has a wider range of characteristic impedance and a wider operating frequency band [36].

The antenna was developed using design parameters that were calculated from the wavelength ($\lambda_{L}$) corresponding to the lower band-edge frequency of UWB, which is approximately 2.3 GHz. The approximate range of R1 and L3 can be determined using Equation (1):

(1) $$\frac{\lambda_{L}}{8}\leq 2R_{1}+L_{3}\leq \frac{\lambda_{L}}{4}\;$$

The thickness of the substrate can be determined using Equation (2):

(2) $$H\approx 0.01\lambda_{L}$$

**Figure 8:**
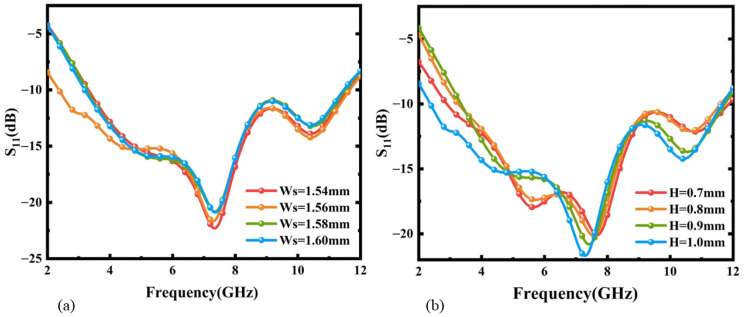
(**a**) The influence of the feedline Ws on antenna S11. (**b**) The influence of the substrate thickness H on antenna S11.

The influence of parameters such as the width Ws of the antenna feedline, the width L1 of the ground plane, the radius R2 of the semi-circular slot, and the thickness H of the substrate on the impedance bandwidth was analyzed using the simulation software Ansys HFSS 2020. Agrawall N P once pointed out in his research that the distance g between the CPW ground plane and the feed point has a significant impact on the antenna impedance bandwidth [37], and this influence is mainly reflected in the upper limit frequency. Keeping the other parameters constant, a simulation analysis was conducted on the feedline width Ws (when the width Wd of the ground plane gap is unchanged, the distance g between the CPW ground plane and the feed point increases as Ws decreases). The results are shown in Figure 8a. In the low-frequency band, when Ws = 1.56 mm, the antenna impedance bandwidth increases with an increase in Ws (i.e., as the distance g decreases, the impedance bandwidth increases). This verifies the research conducted by Agrawall N P. To maintain a wide impedance bandwidth for the antenna, according to the simulation results in Figure 8a, Ws = 1.56 mm can be selected.

By varying the thickness H of the substrate within a certain range, S11 was obtained. The results are shown in Figure 8b. It can be seen from the comparison that the influence of the substrate thickness H on the antenna impedance bandwidth is more intense in the low-frequency band and tends to stabilize in the high-frequency band. To keep the overall return loss of the antenna at a low level, based on the simulation results, H = 1 mm can be selected. The final design dimensions are summarized in Table 1.

#### 2.3.1. Surface Current Distribution

**Figure 9:**
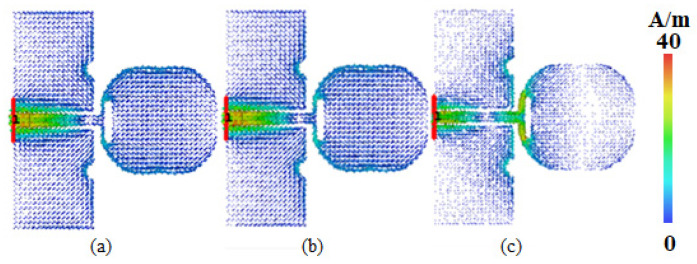
Surface current distributions at (**a**) 5.25 GHz; (**b**) 5.75 GHz; (**c**) 8 GHz.

Figure 9 shows the surface current distribution of the antenna at three frequency points of 5.25 GHz, 5.75 GHz, and 8 GHz. It can be seen that the surface current is mainly concentrated near the semicircular slot on the feeder, radiation patch edge, and grounding plane, and the maximum currents are 36 A/m, 37.5 A/m, and 39.4 A/m, respectively.

#### 2.3.2. Radiation Efficiency and Peak Gain

**Figure 10:**
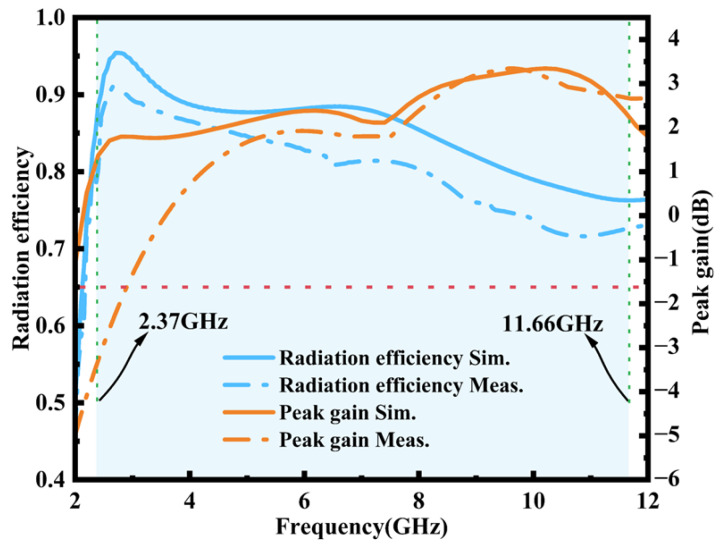
Simulated and measured results of the radiation efficiency and peak gain for the proposed antenna.

Figure 10 presents the peak gain versus frequency for the antenna; it can be seen that the simulated peak gain increases with an increase in frequency, and the antenna gain is greater than zero throughout the entire UWB operating frequency band (2.3~11.66 GHz). The simulated peak gain reaches a minimum of 1.24 dB at 2.37 GHz and a maximum of 3.34 dB at 10.27 GHz. In addition, the frequency versus radiation efficiency relationship in the figure shows that the simulated radiation efficiency is more than 75% throughout the operating bandwidth, with an average radiation efficiency of about 84.46%. The simulated radiation efficiency shows a decreasing trend with increasing frequency, which is due to the fact that the patch size becomes larger than the corresponding wavelength as the frequency increases [38,39,40]. The antenna was attached to the chest to test its performance during human wearing. In the actual test, the measured values of the antenna’s radiation efficiency and peak gain decreased to varying degrees, as shown in Figure 10. The reason for the significant decrease in the measured radiation efficiency in the 6–12 GHz range is that the human body, as a lossy medium, absorbs electromagnetic energy, especially in the high-frequency band. Due to the significant resonance absorption effect of water molecules, the radiation efficiency decreases.

#### 2.3.3. Fabrication of Antenna

**Figure 11:**
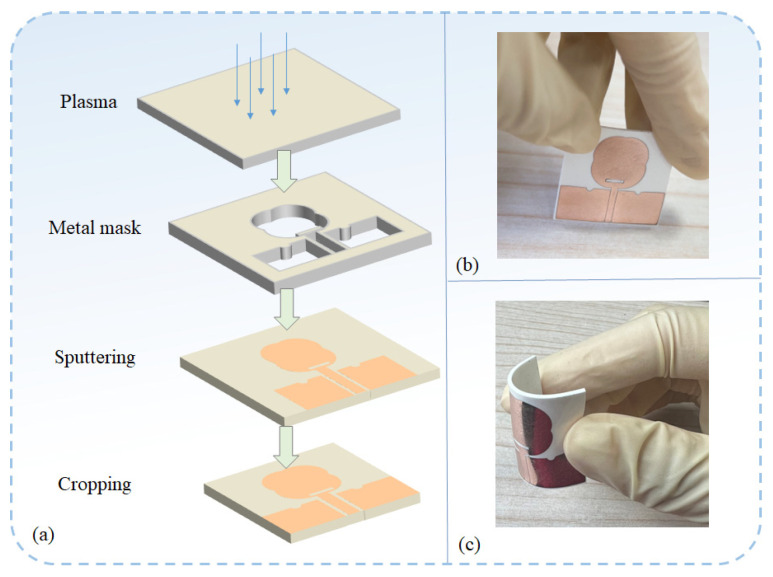
(**a**) Flow chart of antenna manufacturing process. (**b**) Front view of antenna plane state. (**c**) Side view of antenna bending state.

In this work, a sputtering process was used to fabricate the antenna, and the overall manufacturing flow chart is shown in Figure 11a. The composite substrate exhibited hydrophobic properties, which could reduce the adhesion effect between the structures. Therefore, an oxygen plasma degumming process was first used to treat the substrate surface to improve its adhesion to copper. Then, a mask was placed on the flexible composite substrate, and the thickness of the copper film sputtered by the magnetron sputtering machine was determined according to the skin effect. Finally, the excess part of the substrate was cut off. The antenna is physically shown in Figure 11b,c.

## 3. Results and Applications

### 3.1. Antenna Frequency Test

**Figure 12:**
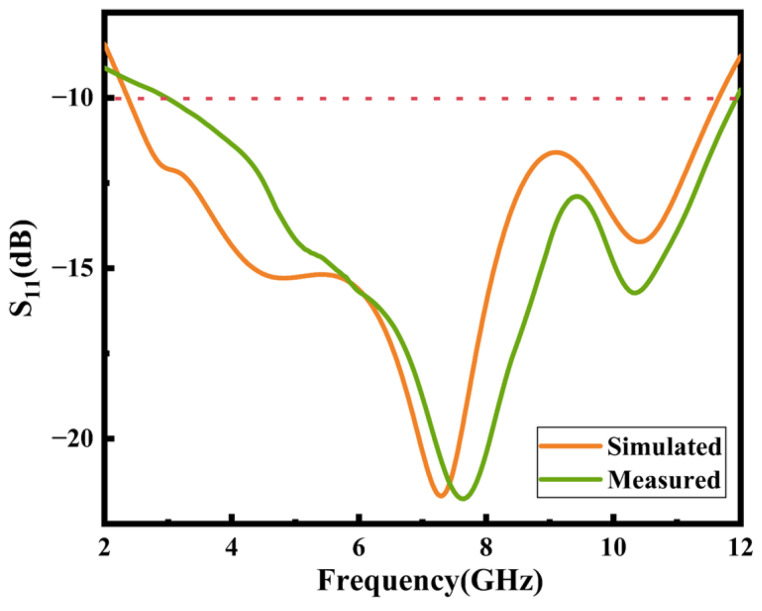
Return loss S11 test results for invariant state.

The measured callback loss S11 when the antenna is not deformed and is in a natural state is shown in Figure 12. The simulation results show that when the antenna is not bent, the −10 dB frequency bandwidth of the antenna is 9.57 GHz. Although S11 is shifted to the high-frequency band by about 0.35 GHz in the test results, it is basically in line with the simulation curve and covers the frequency band of 2.37 GHz to 11.66 GHz with a relatively good frequency characteristic. The errors in the simulated and tested return loss curves may originate from the unstable dielectric constant due to the lack of homogeneous mixing in some areas during the processing of the substrate. Secondly, machining errors in the substrate size and the ohmic loss caused by SMA head welding will also have had a certain impact on the result.

**Figure 13:**
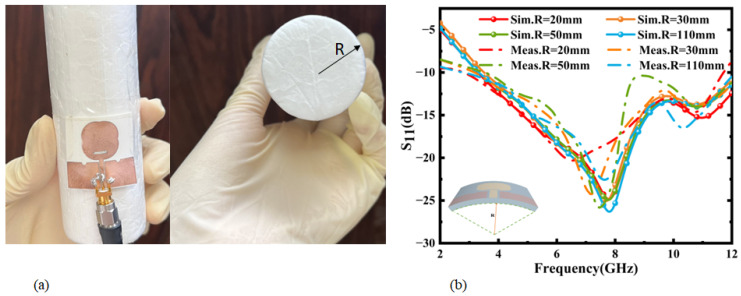
(**a**) Antenna conformal test and physical view of cylindrical foam. (**b**) Radiation performance test of antenna: return loss of antenna along y-axis for different bending radii.

Since the antenna is to be integrated into a garment, it will be difficult for the antenna to remain flat; therefore, its performance under bending conditions must be evaluated. The antenna was taped to cylindrical foam as shown in Figure 13a, and the antenna was tested for bending in the y-axis direction. The reason for choosing the foam was that its dielectric constant was closer to that of air. Figure 13b shows the return loss of the antenna along the y-axis for cylinders of different radii. It can be seen from the simulation results that with the change in the bending radius, the frequency point of the antenna shifted slightly, and the antenna maintained good performance. The measured results show that the bandwidth of the antenna was reduced by 5%, 14%, and 12% of the bandwidth when the antenna was on cylindrical foam with radii of 30 mm, 50 mm, and 110 mm, respectively, as compared to transmitting under flat conditions. Therefore, the antenna radiation is not proportional to the bending degree. The operating bandwidth of the antenna changes with different degrees of bending, and the function of ultra-wideband transmission can be realized at a certain degree of bending.

### 3.2. Radiation Performance Test of Antenna

**Figure 14:**
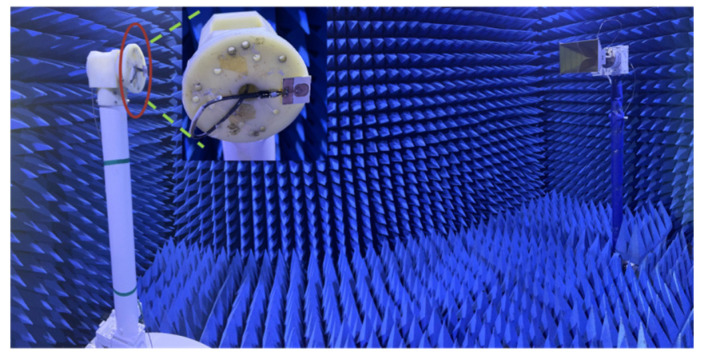
Microwave darkroom test chart.

Figure 14 shows the effect of measuring the far-field radiation pattern of an antenna in a microwave darkroom, where the antenna under test is fixed on a turntable as a receiver and a 2–18 GHz standard gain horn antenna is used as a transmitting antenna.

**Figure 15:**
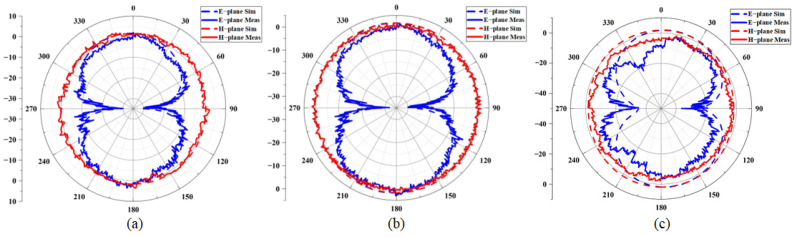
Radiation performance of the antenna at different frequencies: (**a**) 5.25 GHz; (**b**) 5.75 GHz; (**c**) 8 GHz.

**Figure 16:**
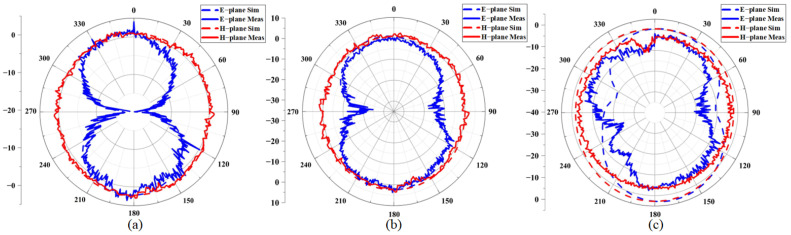
Radiation performance of the antenna at different frequencies, R = 30 mm bending condition: (**a**) 5.25 GHz; (**b**) 5.75 GHz; (**c**) 8 GHz.

Figure 15a–c shows the simulated and measured direction diagrams of the antenna at three frequency points under the invisible variable state. It can be seen that the measured radiation direction diagram is basically consistent with the simulation results, there is no obvious distortion on the E-plane or the H-plane, and the antenna achieves omnidirectional radiation on the H-plane. As shown in Figure 16a–c, the radiation performance of the antenna under a bending radius of 30 mm demonstrates a slight performance degradation at 8 GHz. The antenna may show performance degradation in the high-frequency region, but it can be ignored in practical applications.

### 3.3. Heating Performance of Antenna

**Figure 17:**
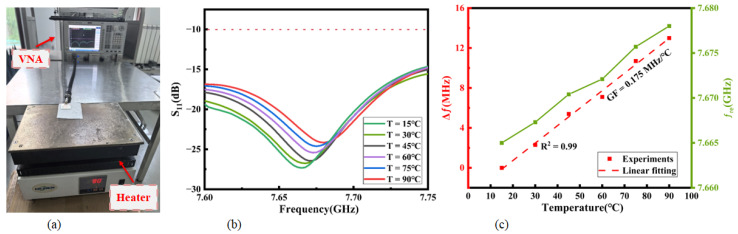
(**a**) The temperature sensing test platform. (**b**) Return loss changes for the antenna from 15 °C to 90 °C. (**c**) GF of the frequency shift versus the corresponding temperature variation and resonant frequency variation.

The antenna was positioned on a heater for thermal treatment as illustrated in Figure 17a; its surface temperature was elevated from 15 °C to 90 °C. A notable frequency shift near the resonant regime exhibited exceptional linearity (R^2^ = 0.99), revealing temperature-dependent resonant characteristics for $GF=\frac{∆f}{\delta T}=0.175\;MHz/{}^{\circ}C$ in Figure 17b,c. The antenna’s superior temperature sensing performance at the resonance frequency suggests promising potential for wearable integration, particularly in scenarios requiring real-time thermal monitoring and adaptive electromagnetic responses. This thermal–frequency correlation mechanism could facilitate innovative co-design strategies for multifunctional wearable devices combining sensing and communication capabilities.

**Figure 18:**
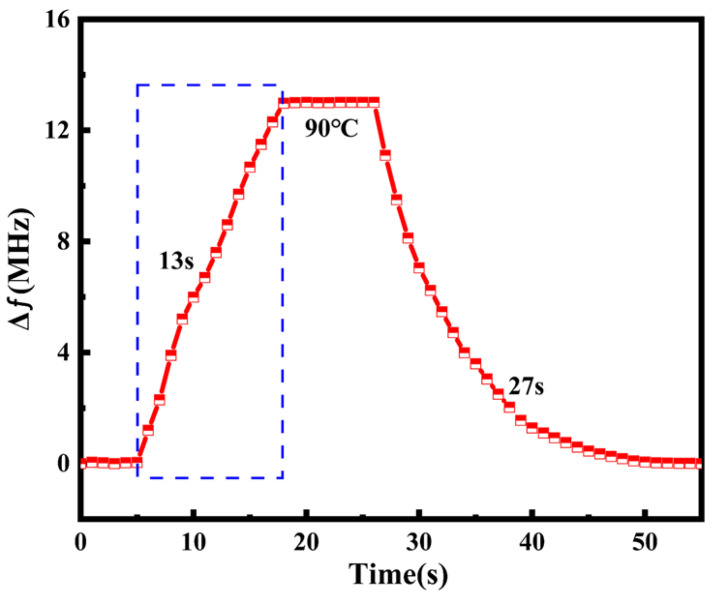
Rapid response and recovery of antenna upon heating and cooling.

The antenna was rapidly moved from a state of 15 °C to a constant-temperature heater at 90 °C. The change in the resonant frequency of the antenna compared to that at the initial temperature is shown in Figure 18. The experiment indicated that its response time was 13 s, as shown by the blue dashed square in Figure 18.

**Figure 19:**
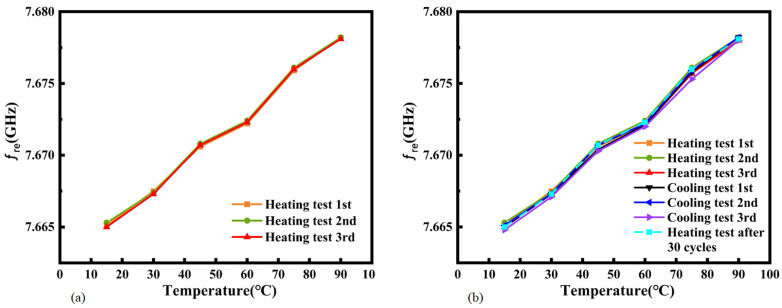
(**a**) Frequency–temperature curves during three heating cycles. (**b**) Thermal sensitivity after repeated heating and cooling between 15 °C and 90 °C.

Three cyclic repeatability tests were carried out on the proposed antenna in order to verify the reliability of the thermal sensitivity. The frequency–temperature curves during the three temperature rise processes were studied and are shown in Figure 19a. It can be seen from the figure that the coincidence of the resonant frequency points in the three experiments was relatively good. When the temperature rose from 15 °C to 90 °C, according to the repeatability error formula of the sensor, the repeatability error ${\bar{y}}_{FS}$ was calculated as (0.0131 + 0.0129 + 0.013)/3 = 0.013. The frequency–temperature curves of the three repeated temperature rise–drop tests are shown in Figure 19b. Hysteresis can be observed between the heating and cooling branches, and the heating and cooling curves do not completely overlap. The maximum deviation ${∆}_{max}$ of the resonant frequency corresponding to the same temperature test point during the heating and cooling processes in the cycle test reached 0.0004. The hysteresis of the thermal sensitivity $e_{H}$ is as follows:

(3) $$e_{H}=\pm \;\frac{{∆}_{max}}{{\bar{y}}_{FS}}=\frac{0.0004}{0.013}=3.07\%$$

The proposed antenna was then subjected to 30 consecutive heating and cooling cycles (varying from 15 °C to 90 °C) to verify the long-term stability of the thermal sensitivity. The new curve after these cycles is shown in Figure 19b. Excellent long-term stability of the thermal sensitivity was demonstrated.

### 3.4. Antenna Performance on Body Models

Since various organs in the human body are lossy media [41], when exposed to the electromagnetic waves radiated by the wearable antenna, the electromagnetic field in the body will generate currents [42], which will absorb and dissipate electromagnetic energy. Therefore, it is necessary to consider whether the wearable antenna can satisfy normal communication requirements when worn on the human body. Secondly, because the antenna transmits radiation, the SAR value should be used to determine whether it is harmful to human tissues [43].

**Figure 20:**
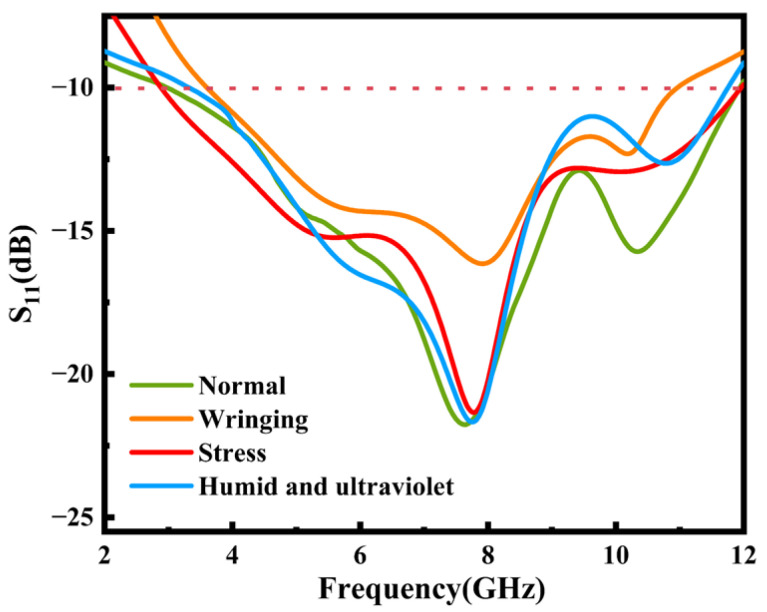
Return loss S11 test results for special states in wearable applications.

The return loss in special situations was tested in order to verify the practicability of the proposed antenna for real-world wearable applications. The antenna was attached to the inner wall of a beaker containing 4% NaCl solution to simulate the humid environment after human sweating. At the same time, a 38 W ultraviolet lamp was turned on, and a slight shift in its resonant frequency occurred after 168 h as shown in Figure 20. The observed phenomenon may be attributed to moisture-induced alterations in the surface conductivity of the antenna, potentially resulting from atmospheric water molecule adsorption. This physicochemical interaction could modify the interfacial dielectric properties, thereby compromising the antenna’s impedance matching efficiency through changes in its surface current distribution and near-field electromagnetic characteristics. In addition, the extreme environmental working state of the antenna when it is completely soaked was simulated by adding a 3 mm thick water film on the surface of the antenna. Due to the strong coupling between the antenna radiation and the high-loss water medium, the return loss curve of the antenna was significantly distorted. Consequently, substantial scattering occurred in the antenna radiation.

Furthermore, to verify the performance of the antenna under cyclic stress, a press machine was used to conduct a 30-cycle longitudinal extrusion experiment on the antenna in a flat state with a force of 2 N to simulate extrusion deformation during the wearing process. Additionally, 300 cycles of transverse manual bending experiments were carried out. The measured return loss after the stress experiment is shown in Figure 20. The shift in the resonant frequency is mainly due to the change in port impedance matching of the antenna under cyclic stress.

**Figure 21:**
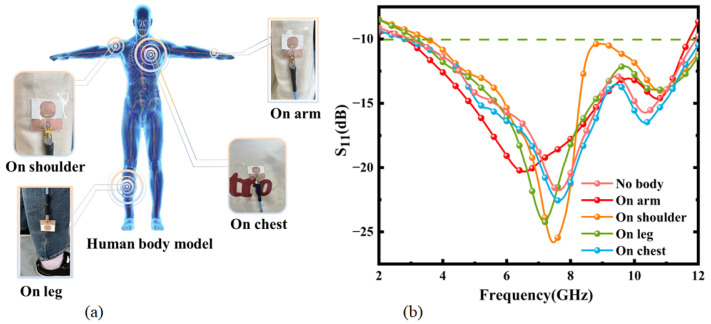
Antenna test: (**a**) Chart of different parts of the human body. (**b**) Measured return loss.

The antenna was fixed on the arm, shoulder, thigh, and chest of the human body using transparent tape as shown in Figure 21a for testing, and the final measured return loss results are shown in Figure 21b. The antenna was least affected when it was placed on the chest, and the resulting curve fit well with the return loss curve before wearing. When it was placed on the arm, with the largest curvature radius, only 0.48 GHz frequency deviation was generated at 2.37 GHz in the low-frequency range, and the change was lesser in the high-frequency range, which made the overall performance better. In addition, the working band of the antenna shifted due to the human body’s obstruction and scattering affecting the communication quality of the antenna during the actual measurement process. Based on the results obtained in the simulations and lab experiments, bending of the antenna, which varies with the part of the body on which the antenna is worn, does not undermine the effectiveness of the proposed wearable antenna for temperature sensing. The application of this proposed antenna on the chest will be the main direction of our future research and exploration.

**Figure 22:**
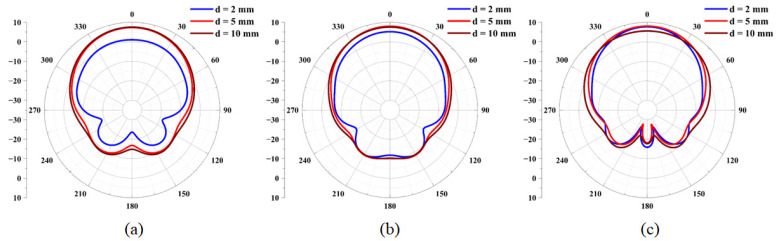
Radiation patterns of the antenna in the E-plane for various distances at frequencies of (**a**) 5.25 GHz, (**b**) 5.75 GHz, and (**c**) 8.0 GHz.

In wearable communications, when antennas are attached to clothing, the distance between the antenna and the human body during movement may continuously vary, thereby altering the radiation pattern characteristics of the antenna. To investigate this effect, the antenna was positioned at distances of 2 mm, 5 mm, and 10 mm from the skin layer, and the corresponding radiation patterns were recorded at different operating frequencies, as illustrated in Figure 22. The radiation patterns of the antenna demonstrate notable stability across these varying distances. Furthermore, the influence of the human body on the antenna’s radiation gradually diminished as the separation between the antenna and the human skin increased. The radiation patterns tended to stabilize and maintain a symmetrical configuration under these conditions.

**Figure 23:**
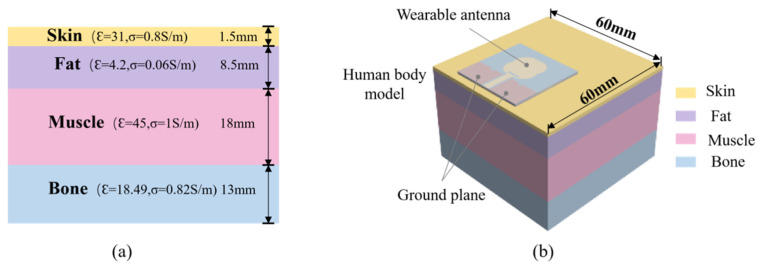
(**a**) The four-layer human tissue structure model. (**b**) The position relationship between the body tissue and the antenna.

**Figure 24:**
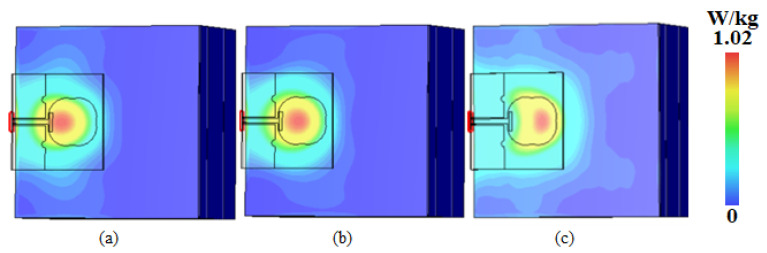
SAR simulation test plots at (**a**) 5.25 GHz, (**b**) 5.75 GHz, and (**c**) 8 GHz.

As for the SAR value for the antenna worn on the human body, the electromagnetic simulation software CST Studio Suite 2022 was used in this paper to study it, and a four-layer human tissue model (skin, fat, muscle, and bone from top to bottom) was created at a position 3 mm below the antenna, as shown in Figure 23. The electromagnetic characteristic parameters of the human tissue were in accordance with the literature when the input power was 0.02 W [44]. The final simulation results are shown in Figure 24, where the peak SARs of 1 g of tissue at 5.25 GHz, 5.75 GHz, and 8 GHz were 1.02 W/kg, 0.602 W/kg, and 0.43 W/kg, respectively.

**Figure 25:**
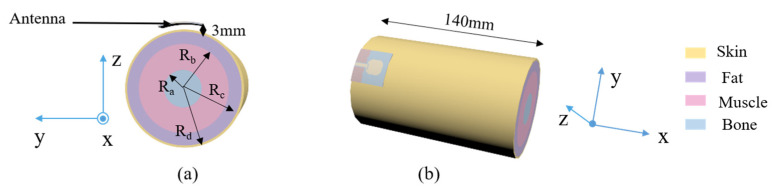
The antenna on top of the arm model: (**a**) cross-sectional view; (**b**) oblique view (R_a_ = 13 mm, R_b_ = 31 mm, R_c_ = 39.5 mm, R_d_ = 41 mm).

**Figure 26:**
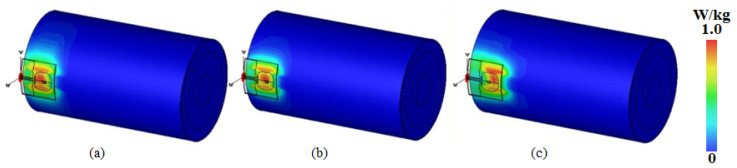
SAR distribution in the body model of the arm at (**a**) 5.25 GHz, (**b**) 5.75 GHz, and (**c**) 8 GHz.

In addition, the SAR distribution was further analyzed by simulating the antenna worn on the human arm through a multilayer cylindrical mold, as shown in Figure 25. The simulation results are shown in Figure 26, and the calculated maximum 1 g tissue average SARs were 0.998 W/kg, 0.7 W/kg, and 0.422 W/kg for 5.25 GHz, 5.75 GHz, and 8 GHz, respectively; these are far lower than the FCC limit (1.6 W/kg), and, thus, the designed ultra-wideband antenna is suitable for wearable applications.

### 3.5. Comparison

Table 2 compares the proposed antenna with some previous works, and the results show that the flexible substrate fabricated with doped polymer material in this paper achieves higher radiation efficiency with a smaller size. Also, it shows better performance in terms of its impedance bandwidth.

## 4. Conclusions

An ultra-wideband antenna based on a flexible composite dielectric substrate was proposed, in which the dielectric modulation of the substrate was realized by doping different ratios of fillers into the matrix. The use of silane coupling agent improved the dispersion of filler TiO_2_ in the matrix, which further enhanced the dielectric properties of the substrate and expanded the antenna’s potential for wearable applications. A miniaturized ultra-wideband antenna was designed and machined in order to verify the utility and feasibility of the new substrate. By using a magnetron sputtering process, the adhesion and structural accuracy of the metal structure on the antenna surface were improved. The antenna demonstrated significant potential for future applications in detecting environmental thermal changes. In order to test its usage in a real environment, the radiation performance of the antenna under bending conditions was simulated and tested by means of cylindrical foams with different curvatures. In addition, the safety requirements were satisfied, as the SAR distribution was further analyzed by simulating the antenna worn on the human arm through a multilayer cylindrical mold. Therefore, the antenna, made of a dielectric substrate with higher toughness, a higher dielectric constant, and a lower loss angle tangent, is suitable for ultra-wideband wireless communication, which provides a direction for the integration of antennas with medical devices.

## Figures and Tables

**Table 1:** The final size parameters of the antenna.

Parameter	Value (mm)	Parameter	Value (mm)
L	30	L7	13.5
L1	11	L8	1
L2	22	R1	6
L3	4	R2	1
L4	3	H	1
L5	2.9	Wd	2.3
L6	3.1	Ws	1.56

**Table 2:** Performance comparison of the proposed antenna.

Ref.	Dimensions (mm)	Bandwidth (GHz)	Substrate Used	Radiation Efficiency (%)	Gain (dB)
[13]	170 × 170	2.35–2.45	alumina and barium titanate	-	data
[14]	40.5 × 40	3.3–12	E-glass fiber mat and epoxy resin	94	-
[45]	40 × 30	2.3–5.3	photo paper	80.29	-
[46]	80 × 67	3.7–10.3	PDMS	45	5
[47]	29 × 25	1.73–20	PDMS	40	4.12
Proposed	30 × 30	2.37–10.66	TiO_2_-PTFE-PDMS	95.4	3.34

## Data Availability

The data that support the findings of this study are available from the author (B.M.) upon reasonable request.

## References

[1] Mohamadzade B., Hashmi R.M., Simorangkir R.B.V.B., Gharaei R., Ur Rehman S., Abbasi Q.H. (2019). Recent Advances in Fabrication Methods for Flexible Antennas in Wearable Devices: State of the Art. Sensors.

[2] Hertleer C., Tronquo A., Rogier H., Vallozzi L., Van Langenhove L. (2007). Aperture-Coupled Patch Antenna for Integration Into Wearable Textile Systems. IEEE Antennas Wirel. Propag. Lett..

[3] Khaleel H.R., Al-Rizzo H.M., Rucker D.G. (2012). Compact Polyimide-Based Antennas for Flexible Displays. J. Disp. Technol..

[4] Caldeira J.M., Rodrigues J.J., Lorenz P. (2012). Toward ubiquitous mobility solutions for body sensor networks on healthcare. IEEE Commun. Mag..

[5] Gao G.-P., Hu B., Wang S.F., Yang C. (2018). Wearable Circular Ring Slot Antenna With EBG Structure for Wireless Body Area Network. IEEE Antennas Wirel. Propag. Lett..

[6] Stoppa M., Chiolerio A. (2014). Wearable Electronics and Smart Textiles: A Critical Review. Sensors.

[7] Paracha K.N., Rahim S.K.A., Soh P.J., Kamarudin M.R., Tan K.-G., Lo Y.C., Islam M.T. (2019). A Low Profile, Dual-band, Dual Polarized Antenna for Indoor/Outdoor Wearable Application. IEEE Access.

[8] Zhang K., Soh P.J., Yan S. (2021). Meta-Wearable Antennas—A Review of Metamaterial Based Antennas in Wireless Body Area Networks. Materials.

[9] Xu F., Wei B., Li W., Liu J., Liu W., Qiu Y. (2015). Cylindrical conformal single-patch microstrip antennas based on three-dimensional woven glass fiber/epoxy resin composites. Compos. B.

[10] Al-Sehemi A.G., Al-Ghamdi A.A., Dishovsky N.T., Malinova P., Atanasov N.T., Atanasova G.L. (2021). Natural rubber composites containing low and high dielectric constant fillers and their application as substrates for compact flexible antennas. Polym. Polym. Compos..

[11] Babar A.A., Bhagavati V.A., Ukkonen L., Elsherbeni A.Z., Kallio P., Sydanheimo L. (2012). Performance of high-permittivity ceramic-polymer composite as a substrate for UHF RFID tag antennas. Int. J. Antennas Propag..

[12] Vilesh V.L., Ganesanpotti S. (2022). Silicone Rubber-BaBiLiTeO_6_ Composites: Flexible Microwave Substrates for 5G Applications. J. Electron. Mater..

[13] Catarinucci L., Chietera F.P., Colella R. (2022). Permittivity-Customizable Ceramic-Doped Silicone Substrates Shaped With 3-D-Printed Molds to Design Flexible and Conformal Antennas. IEEE Trans. Antennas Propag..

[14] Elmobarak H.A., Rahim S.K.A., Castel X., Himdi M. (2019). Flexible conductive fabric/E-glass fibre composite ultra-wideband antenna for future wireless networks. IET Microw. Antennas Propag..

[15] Loss C., Gonçalves R., Pinho P., Salvado R. (2019). Influence of some structural parameters on the dielectric behavior of materials for textile antennas. Text. Res. J..

[16] Salvado R., Loss C., Gonçalves R., Pinho P. (2012). Textile Materials for the Design of Wearable Antennas: A Survey. Sensors.

[17] Lilja J., Salonen P., Kaija T., De M. (2012). Design and Manufacturing of Robust Textile Antennas for Harsh Environments. IEEE Trans. Antennas Propag..

[18] Wang Y., Edwards E., Hooper I., Clow N., Grant P.S. (2015). Scalable polymer-based ferrite composites with matching permeability and permittivity for high-frequency applications. Appl. Phys. A.

[19] Koulouridis S., Kiziltas G., Zhou Y., Hansford D.J., Volakis J.L. (2006). Polymer–Ceramic Composites for Microwave Applications: Fabrication and Performance Assessment. IEEE Trans. Microw. Theory Tech..

[20] Liu Y., Wu P., Kang P., Li L., Li Q. (2021). PDMS-based composites with stable dielectric properties at varied frequency via Sr-doped CaCu_3_Ti_4_O_12_ nanowires for flexible wideband antenna substrate. J. Mater. Sci. Mater. Electron..

[21] Dang Z.M., Yuan J.K., Zha J.W., Zhou T., Li S.T., Hu G.H. (2011). Fundamentals, processes and applications of high-permittivity polymer-matrix composites. Prog. Mater. Sci..

[22] Ivanova R., Kotsilkova R., Ivanov E., Donato R.K., Silvestre C. (2020). Composition dependence in surface properties of poly(lactic acid)/graphene/carbon nanotube composites. Mater. Chem. Phys..

[23] Zhang Y., Huo P., Wang J., Liu X., Rong C., Wang G. (2013). Dielectric percolative composites with high dielectric constant and low dielectric loss based on sulfonated poly(aryl ether ketone) and a-MWCNTs coated with polyaniline. J. Mater. Chem. C.

[24] Feng M., Huang Y., Cheng T., Liu X. (2017). Synergistic effect of graphene oxide and carbon nanotubes on sulfonated poly(arylene ether nitrile)-based proton conducting membranes. Int. J. Hydrogen Energy.

[25] Ali N., Ali F., Saeed S. (2019). Structural characteristics and electrochemical properties of sulfonated polyimide claybased composite fabricated by a solution casting method. J. Mater. Sci. Mater. Electron..

[26] Nguyen T.C., Nguyen T.D., Vu D.T., Dinh D.-P., Nguyen A.-H., Ly T.-N., Dao P.-H., Bach L.-G., Thai H. (2020). Modification of titanium dioxide nanoparticles with 3-(trimethoxysilyl)propyl methacrylate silane coupling agent. J. Chem..

[27] Vasudevan S., Fullerton-Shirey S.K. (2019). Effect of nanoparticle shape on the electrical and thermal properties of solid polymer electrolytes. J. Phys. Chem. C.

[28] Thomason J. (2020). A review of the analysis and characterisation of polymeric glass fibre sizings. Polym. Test..

[29] Zindani D., Kumar K. (2019). An Insight into Additive Manufacturing of Fiber Reinforced Polymer Composite. Int. J. Lightweight Mater. Manuf..

[30] Wang C., Mao H., Wang C., Fu S. (2011). Dispersibility and Hydrophobicity Analysis of Titanium Dioxide Nanoparticles Grafted with Silane Coupling Agent. Ind. Eng. Chem. Res..

[31] Cheng Q., Li C., Pavlinek V., Saha P., Wang H. (2006). Surface-modified antibacterial TiO_2_/Ag+ nanoparticles: Preparation and properties. Appl. Surf. Sci..

[32] Sharma P.K., Chung J.Y. (2023). Evaluation of polydimethylsiloxane (PDMS) as a substrate for the realization of flexible/wearable antennas and sensors. Micromachines.

[33] Kundu S., Chatterjee A., Jana S.K., Parui S.K. (2018). A Compact Umbrella-Shaped UWB Antenna with Gain Augmentation Using Frequency Selective Surface. Radioengineering.

[34] Zhang J., Cao P., Huang Y., Alrawashdeh R., Zhu X. (2014). Compact planar ultra-wideband antenna with quintuple band-notched characteristics. Microw. Antennas Propag. Lett..

[35] Hertleer C., Rogier H., Vallozzi L., Van Langenhove L. (2009). A textile antenna for off-body communication integrated into protective clothing for firefighters. IEEE Trans. Antennas Propag..

[36] Hassan A., Ali S., Hassan G., Bae J., Lee C.H. (2017). Inkjet-printed antenna on thin PET substrate for dual band Wi-Fi communications. Microsyst. Technol..

[37] James N.K., Jacob K.S., Bae J., Lee C.H. (2010). Ba(Mg_1/3_Ta_2/3_)O_3_ filled ptfe composites for microwave substrate applications. Mater. Chem. Phys..

[38] Saha T.K., Knaus T.N., Khosla A., Bae J., Lee C.H. (2022). A CPW-fed flexible UWB antenna for IoT applications. Microsyst. Technol..

[39] Chaouche Y.B., Nedil M., Messaoudene I., Bae J., Lee C.H. CPW-fed Hexagonal Modified Sierpinski Carpet Fractal Antenna for UWB Applications. Proceedings of the IEEE International Symposium on Antennas and Propagation and USNC-URSI Radio Science Meeting.

[40] Saha T.K., Goodbody C., Karacolak T., Sekhar P.K. (2019). A compact monopole antenna for ultra-wideband applications. Microw. Opt. Technol. Lett..

[41] Abirami B.S., Sundarsingh E.F. (2017). EBG-Backed Flexible Printed Yagi-Uda Antenna for On-Body Communication. IEEE Trans. Antennas Propag..

[42] Li J. Computer Aided Modeling and Simulation of Cooling Fan System. Proceedings of the 2011 International Symposium on Information Engineering and Electronic Commerce (IEEC2011).

[43] Li E., Li X.J., Seet B.-C. (2021). A Triband Slot Patch Antenna for Conformal and Wearable Applications. Electronics.

[44] Zhang R., Liu J.W., Wang Y.Y., Luo Z., Zhang B., Duan J. (2021). Flexible Wearable Composite Antennas for Global Wireless Communication Systems. Sensors.

[45] Jabbar A., Zubair M., Naveed M.A., Mehmood M.Q., Massoud Y. (2022). A photopaper-based low-cost, wideband wearable antenna for wireless body area network applications. IET Microw. Antennas Propag..

[46] Simorangkir R.B.V.B., Kiourti A., Esselle K.P. (2018). UWB Wearable Antenna With a Full Ground Plane Based on PDMS-Embedded Conductive Fabric. IEEE Antennas Wirel. Propag. Lett..

[47] Zhang C.Y., Guo J.Q., Lian S.L., Chi Z.T., Sun Z., Zheng Y., Sun B., Liu T. (2023). A flexible and transparent pliers shaped antenna for ultra-wideband applications. Flex. Print. Electron..

